# *POT1* genetic testing in melanoma-prone families in Sweden: germline variant prevalence and tumor spectrum in identified carriers

**DOI:** 10.2340/1651-226X.2025.44048

**Published:** 2025-08-25

**Authors:** Konstantinos Papadakis, Francesca Portelli, Karina Schultz, Hedvig Olsson Sterky, Ismini Vassilaki, Jan Lapins, Michael R. Sargen, Sofia Obolenski, David J. Adams, Muyi Yang, Veronica Höiom, Hildur Helgadottir

**Affiliations:** a Department of Oncology and Pathology, Karolinska Institutet, Stockholm, Sweden; b Department of Clinical Pathology and Cancer Diagnostics, Karolinska University Hospital, Stockholm, Sweden; c Theme Cancer, Karolinska University Hospital, Stockholm, Sweden; d Dermatology and Venereology Unit, Department of Medicine Huddinge, Karolinska Institutet, Stockholm, Sweden; e Department of Dermatology, Karolinska University Hospital, Stockholm, Sweden; f Division of Cancer Epidemiology and Genetics, National Cancer Institute, National Institutes of Health, Rockville, Maryland, USA; g Experimental Cancer Genetics, Wellcome Sanger Institute, Wellcome Trust Genome Campus, Hinxton, Cambridge, UK; h Department of Dermatology, Leiden University Medical Centre, Leiden, The Netherlands

**Keywords:** POT1, cutaneous melanoma, sarcoma, cancer, telomere, mutation, pathogenic variant

## Abstract

**Background and purpose:**

Approximately 5–10% of cutaneous melanoma occurs in individuals with a family history of the disease. While known high-penetrance genes, such as *CDKN2A*, explain some cases, a substantial proportion of hereditary melanoma remains genetically undefined. Recently, germline variants in genes involved in telomere regulation, including *POT1*, *TERT*, *ACD*, and *TERF2IP*, have been identified in melanoma-prone families. This study investigated the prevalence and pathogenicity of *POT1* variants in a Swedish familial melanoma cohort.

**Patient/material and methods:**

A total of 168 familial melanoma cases were screened for *CDKN2A*, *CDK4*, *BAP1*, and *POT1*. The population frequency of pathogenic variants (PVs) was assessed using the SweGen and the gnomAD databases. Functional evaluation was performed using a saturation genome editing (SGE) assay. Telomere length analysis was performed using quantitative polymerase chain reaction (qPCR) on blood-derived DNA from melanoma patients and healthy controls. The melanomas of the carriers were reviewed by expert dermatopathologists.

**Results:**

Among the 161 *CDKN2A/CDK4/BAP1*-negative melanoma families included in this cohort, only one likely PV in *POT1* (c.676C > A, p.His226Asn) was identified (0.6%). Population data confirmed its rarity. The carrier family exhibited multiple early-onset melanomas, with two out of three invasive cases displaying spitzoid morphology, and several other tumors. No significant telomere length differences were observed between carriers and controls. Two additional *POT1* variants of uncertain significance were detected; both were predicted to be benign.

**Interpretation:**

*POT1* PVs were rare in the studied Swedish familial melanoma cases, implying limited contribution to hereditary melanoma in this population. Nonetheless, the identification of a previously unknown likely PV further supports the need for continued genetic screening in selected cases. *POT1* testing should be considered in families with multiple melanomas, early onset and spitzoid histopathology, and co-occurring with other syndromic tumors.

## Introduction

It is estimated that 5–10% of cutaneous malignant melanoma (in this article referred to as melanoma) occurs in individuals with a family history of the disease. Since only around 10–30% of familial melanoma cases are attributed to known germline aberrations in established high-penetrance genes, principally *CDKN2A*, a significant amount of the hereditary risk for melanoma is still largely undefined [1–4]. More recently, the focus has centered on germline variants in genes implicated in telomere dysregulation, including *POT1*, *TERT*, *ACD*, and *TERF2IP*, as a novel pathway underlying familial melanoma [5–9]. Telomeres are regions at the ends of linear chromosomes composed of repeating nucleotide sequences, shielding the chromosome termini from enzymatic degradation and aberrant DNA recombination during cell division. Telomeres progressively shorten with each round of cell division – an essential phenomenon in the replicative lifespan of cells and the aging process. To preserve telomere length and ensure genomic stability, the telomerase enzyme synthesizes new telomeric repeats with every cycle [10]. Among telomere regulating genes, germline pathogenic variants (PVs) in *POT1* have been found at the highest frequencies in melanoma-prone families – up to 9% – though with considerable variation across different cohorts [5, 6, 9, 11, 12].

The *POT1* (protection of telomeres 1) gene encodes a protein-key component of shelterin, a telomere-specific protein complex, consisting of six subunits, which is responsible for protecting telomeres and maintaining their length. POT1 subunit binds to the single-stranded 3’ telomeric DNA (ssDNA) overhang, enabling the whole shelterin complex to mediate and regulate the interaction of telomerase with telomeres [13, 14]. Therefore, protein-altering variants within this complex can dysregulate shelterin’s function in protecting telomeres from being inappropriately processed by the DNA-repair machinery. Germline genome sequencing studies have revealed several *POT1* PVs that alter the protein’s capacity to bind to ssDNA, resulting in elongated and aberrant telomeres. These abnormalities ultimately promote carcinogenesis and predispose individuals to a familial cancer syndrome that, besides melanoma [5, 6, 15], includes glioma [16], chronic lymphoblastic leukemia [17], lymphoid and myeloid neoplasms [18], colorectal cancer [19], and angiosarcoma [20, 21]. In this article, we report the results of *POT1* germline genetic testing that has been conducted in Stockholm, Sweden, as part of the routine gene panel testing for hereditary melanoma in families with melanoma and cancer susceptibility. We further report on the tumor spectrum and the histopathological features of the melanoma cases reviewed in identified carriers.

## Patients/material and methods

### Patients and families

Since 2021, all identified melanoma families in Stockholm have undergone *POT1* testing, as it has been implemented in routine gene panel testing for familial melanoma (including also *CDKN2A*, *CDK4*, and *BAP1*). The Stockholm region is currently (2025) the only region in Sweden that has implemented *POT1* in the routine gene panel for familial melanoma. For this testing, familial melanoma has been defined as three or more cases of melanoma (invasive or in situ) in a family, or two first-degree relatives (FDRs) with melanoma if one is diagnosed before the age of 55. At the Hereditary Melanoma Clinic, Karolinska University Hospital, genetic testing was offered to affected members of melanoma-prone families fulfilling the criteria. Melanoma diagnoses in families were verified by pathology or clinical records. This study was approved by the Swedish Ethical Review Authority (Dnr. 2012/1192-32 and 2018/803-31).

### POT1 germline genetic testing in melanoma families

The *POT1* germline genetic screening was performed as a clinical test at an accredited laboratory, Karolinska University Laboratory. The method used was accredited, with a library preparation performed using Ion AmpliSeq™ technology, followed by sequencing on Ion S5™ system, and data analysis using Ion Reporter™ software (Life Technologies). All *POT1* exons and exon-intron boundaries were covered by the analysis. Only gene variants with a minor allele frequency (MAF) of less than 0.01 were considered. The clinical variant interpretation guidelines from the American College of Medical Genetics and Genomics (ACMG) and Association for Molecular Pathology (AMP) were used to assess whether a variant was (likely) pathogenic, of uncertain significance (VUS), or (likely) benign [22]. ClinVar and the VarSome databases were used for variant classification [23, 24]. In parallel, data from saturation genome editing (SGE) were used to functionally assess the impact of *POT*1 germline variants. This high-throughput approach introduces all possible single-nucleotide variants into a genomic region of interest and measures their functional consequences in a pooled assay. The method was performed as described by Obolenski et al. [25].

### POT1 variant detection in normal population

To explore the frequency of *POT1* PVs/likely PVs in the general population, we used the SweGen Variant Frequency Dataset [26] and non-Finnish Europeans (NFE) from the gnomAD v4.1.0 [27] as a control cohort. The SweGen dataset consists of genome variant frequencies for 1,000 Swedish individuals, representing a cross-section of the Swedish population. The gnomAD v4.1.0 dataset consists of exome data from over 800,000 individuals, whereof 590,031 are of NFE descent. MAFs were used to assess the population frequency of variants, with data obtained from SweGen, NFE, and gnomAD.

### Telomere length analyses

Quantitative polymerase chain reaction (qPCR) was used to measure the relative telomere length using a protocol that was adapted from Joglekar et al. [28]. Briefly, the analyses were done using PowerUp™ SYBR™ Green Master Mix (Applied Biosystems), forward and reverse primers for the telomere gene and the house-keeping gene, human β-globin (HBG), respectively, and genomic DNA extracted from blood samples. The qPCR reactions were then conducted using the QuantStudio™ 7 Flex Real-Time PCR System (Applied Biosystems). All samples were run in triplicates. Relative telomere length was determined for 74 melanoma patients (53% from familial cases, including members of a family identified with a *POT1* PV) and controls from the general population (blood donors). Relative quantification of gene expression was calculated using the double delta Ct (ΔΔCt) method, with the addition of between-plate control samples, as follows: 2^([ΔΔCt of Tel sample] – [average ΔΔCt HBG-samples]). Univariate analysis of variance in IBM SPSS statistics was used to assess differences in relative telomere length using a linear regression method (general linear model [GLM]). All measurements were adjusted for age and sex.

### Melanoma pathological assessments

Histological slides of the available melanoma cases were reviewed by three pathologists with expertise in melanocytic tumors. Spitzoid morphology was defined, as previously described, by the presence of large spindled and/or epithelioid melanocytes in variable proportions, with absent to minimal pigment and prominent nucleoli [29–31]. In line with previous studies on melanomas in *POT1* variant carriers [32, 33], tumors were classified as having spitzoid morphology if more than 25% of tumor cells exhibited these features. The histological subtype and the percentage of tumor with spitzoid morphology were reported. In some cases, BRAF V600E (VE1) immunohistochemistry (Ventana) was performed as part of the routine workup, using adequate controls on a Ventana BenchMark ULTRA automated staining system. In one case, NGS using Oncomine™ Focus Assay (Thermo Fisher Scientific, USA) was performed.

## Results

### Incidence of POT1 PVs in melanoma families and in the normal population

**Figure 1:**
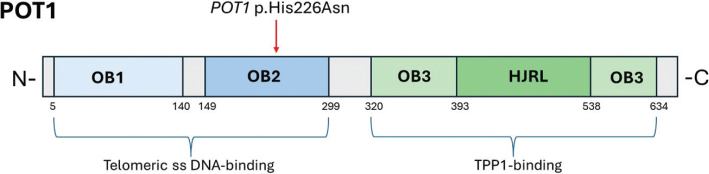
Schematic view of human POT1 protein functional domains. There are three oligonucleotide/oligosaccharide-binding (OB) fold domains, namely, OB1–3, with an embedded Holliday junction resolvase-like (HJRL) domain in the OB3 domain. The location of the likely pathogenic missense variant is indicated by the red arrow.

In total, 168 families fulfilling the definition of familial melanoma were tested for *CDKN2A*, *CDK4*, *BAP1*, and *POT1.* The median age at the earliest melanoma diagnosis in families was 40 years (range 12–78 years), 60% of families had ≥3 cases of melanoma, and 40% had multiple primaries. Seven families were identified with *CDKN2A* mutations, and none with *CDK4* or *BAP1* mutations*.* Only one family with a likely PV was identified; hence, the frequency of *POT1* PVs in this familial melanoma cohort was 0.6%. The *POT1* variant (NM_015450.3:c.676C > A, His226Asn), identified in the family, is a missense variant located in the region encoding the highly conserved oligonucleotide/oligosaccharide-binding (OB) 2 domain (Figure 1). It was reported as a VUS in Clinvar (variation ID: 1018835). The histidine 226 residue is, however, highly evolutionarily conserved, and individual predictors/meta scores used in the VarSome database asserted that this variant is likely to be disruptive. In the SGE assay, the variant c.676C > A was found not to affect cell depletion; however, the c.676_678delinsAAC:CaT→AaC, leading to the same His266Asn missense variant, showed a weak depletion effect in cells, suggesting a functional abnormality. In the SweGen dataset, the p.His226Asn variant was detected in 1/2,000 alleles (MAF = 0.0005) and in the gnomAD NFE dataset in 1,177,492 alleles (MAF = 6.2 × 10^-7^). It was not detected in any other population in gnomAD. No other *POT1* PVs or likely PVs were described in the Swedish dataset. In the gnomAD NFE dataset, the combined MAF of predicted PV/likely PV was 0.00015.

In our cohort, there were two other potential VUS detected, each in a different family. None of them have been reported in the general population (SweGen or gnomAD). One was a missense variant in exon 6 of the *POT1* gene: c.56 G > T, p.(Gly19Val). This variant was detected in a patient from a melanoma-prone family, with a melanoma in situ diagnosed in the fifth decade of life. There was one additional histopathologically confirmed in situ melanoma, and two melanomas without histopathological confirmation, among other family members. The 19 amino-acid position is not well conserved, and the variant is predicted to be benign by individual predictors/meta scores used in the VarSome database. The other variant, –39 G > T, located in the –5’ UTR of *POT1* was observed in a family with three affected members, where the earliest onset occurred in one patient’s fourth decade of life, and one member had multiple primaries. All had invasive melanoma, and the tumors were classified as superficial spreading (SSM) and nodular melanomas (NM). This variant is highly conserved in vertebrate species and may influence splicing; however, in the VarSome prediction databases, it was commonly predicted to be benign. The phenotype of families carrying these two variants, with no other cancer diagnoses in close relatives, was not considered typical of the *POT1* predisposition syndrome either. Furthermore, both these two variants were considered unchanged/neutral in the SGE assay. Hence, neither the c.56 G > T nor the –39 G > T variant was considered pathogenic. The melanomas in these families were therefore not subjected to histopathological review.

### Tumor spectrum in POT1 carrier family

**Figure 2:**
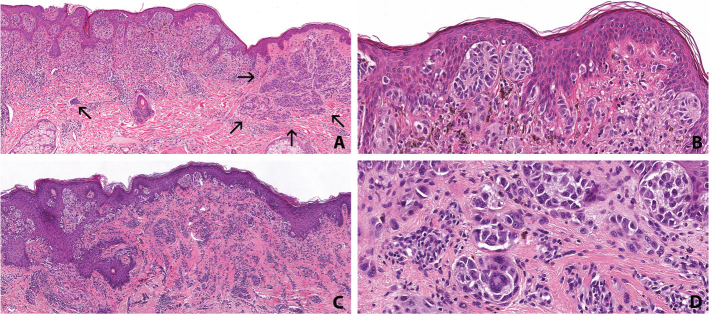
Histopathologic findings of cutaneous melanomas from an individual with a germline variant in POT1 (p.His226Asn). (A) Low magnification of spitzoid melanoma showing epidermal and dermal nests, growing adjacent to a nevus, marked by the arrows (hematoxylin and eosin, original magnification: X4). (B) Spitzoid morphology consisting of atypical enlarged epithelioid melanocytes with abundant eosinophilic cytoplasm. Some of the melanocytes are slightly pigmented (hematoxylin and eosin, original magnification: X20). (C) Superficial spreading melanoma with spitzoid features (hematoxylin and eosin, original magnification: X4). (D) Nests of enlarged spitzoid melanocytes including scattered multinucleated cells embedded in fibrosis (original magnification: X20).

The tumors identified in the family carrying the *POT1* PV are shown in Table 1. To summarize, all three carriers were diagnosed with melanoma, with the earliest onset occurring in the third decade of life in one of the carriers, and multiple primaries were detected. Carrier no.1 was diagnosed with two invasive melanomas. One, located in the head and neck area, showed 100% spitzoid morphology and immunohistochemical reactivity for BRAF V600E, therefore being classified as a spitzoid *BRAF*-mutated melanoma (Figure 2A–B). Breslow score was reported as 0.6 mm, and the tumor was growing adjacent to an intradermal nevus (Figure 2A), suggesting that the melanoma had arisen from a precursor benign lesion. The other, located on the leg, was classified as an SSM with spitzoid features, with 90% of the tumor cells exhibiting spitzoid morphology. This melanoma had a Breslow thickness of 1.2 mm and stained positively for BRAF V600E (Figure 2C–D). Carrier no. 2 was diagnosed with a melanoma in situ on the leg, without spitzoid features, and stained positively for BRAF V600E. Carrier no. 3 was diagnosed, in the eighth decade of life, with a SSM on the leg, without spitzoid features, Breslow 0.6 mm, and an *NRAS* (p.Gln61Arg) mutation detected by NGS analysis. Interestingly, one FDR of a carrier, who tested negative for the *POT1* PV, was diagnosed with an invasive melanoma in the third decade of life. This tumor was classified as an SSM and exhibited only partial (10%) spitzoid morphology. BRAF immunohistochemistry was not performed in this case. There were six additional FDRs from the same family branch that were not genotyped. All six were diagnosed with different types of tumors, including one case of melanoma. In the family, multiple other tumors were reported, including soft tissue tumors (*n* = 5), brain tumor (*n* = 1), and salivary gland tumors (*n* = 2).

**Table 1:** Tumors in family identified with the likely pathogenic variant in POT1.

Family members	No.	Tumors, age of onset (decade)
Carriers	1.	Melanoma (20s), Melanoma (20s)
	2.	Salivary gland tumor (50s), Melanoma in situ (50s), Basal cell carcinoma (50s), Lung carcinoid (60s), Renal angiomyolipoma (70s)
	3.	Melanoma (70s), Tenosynovial giant-cell tumor (70s), Soft tissue sarcoma (70s)
Non-carrier FDR of carrier	1.	Melanoma (20s)
Non-genotyped FDRs of carriers	1.	Melanoma (30s)^†^
	2.	Salivary gland tumor (50s)
	3.	Soft tissue sarcoma (60s)^†^
	4.	Malignant brain tumor (60s)^†^
	5.	Lung cancer (60s)^†^
	6.	Breast cancer (60s), Cerebral cavernous angioma (80s)

FDR: First-degree relative, in the same family branch.

† Deceased from the diagnosed tumor type.

Note: Age of onset is reported by patient’s decade of life (e.g., ‘20s’ refers to the third decade of life, ages 20–29; ‘30s’ to the fourth decade of life, ages 30–39; and so on).

### Telomere length associated with POT1 PV

The adjusted mean telomere lengths in the white blood cells from two of the *POT1* His226Asn variant carriers did not differ significantly from the values obtained from the blood donor control cohort (mean relative expression: 1.180 vs. 1.055, respectively, *p* = 0.645), nor when compared to other melanoma cases (mean relative expression: 1.180 vs 1.546, respectively, *p* = 0.492).

## Discussion and conclusion

### Discussion

Several genetic analysis studies of *POT1* variants have implied their potentially significant role in familial melanoma and various familial cancer syndromes [5, 6, 9, 15–21]. By binding to telomeric ssDNA, as a protein-component of the shelterin complex, POT1 serves a crucial function in preserving telomere integrity [13, 14]. Genetic instability, due to aberrant telomere elongation, resulting in increased risk of carcinogenesis, may arise from dysfunctional variants of *POT1* that disrupt telomerase regulation. For families with a history of melanoma or other related cancers, recent research supports the significance of including *POT1* in genetic screening [5, 6, 15–21].

This study investigated the role of *POT1* germline PVs in familial melanoma within a Swedish cohort. In Stockholm, Sweden, routine genetic testing for familial melanoma, initiated in 2021, revealed the rarity of *POT1* PVs. Out of the 161 families in the familial melanoma cohort, meeting the criteria for melanoma susceptibility testing and being negative for *CDKN2A*, *CDK4*, and *BAP1*, only one family (1/161, 0.6%) was identified carrying a likely pathogenic *POT1* variant. The c.676C > A (p.His226Asn) variant is located in the highly conserved OB2 domain – a region critical for POT1 function – suggesting its disruptive potential to protein’s activity. Given that the variant’s allele frequency in the SweGen dataset was 0.0005 and 1 in 1,610,494 alleles in the gnomAD database, the population-level analysis further confirmed its rarity.

Significant variation in both the prevalence and distribution of *POT1* PVs is determined when comparing across different populations. Compared to our Swedish cohort, 1.8% of melanoma-prone families (4/228) in a Spanish cohort harbored *POT1* PVs, all of which were negative for *CDKN2A/CDK4* mutations [9]. In a multicenter study, *POT1* PVs were also identified in Italian, French, and US cases – with frequencies reaching up to 9% in Italian families, where a founder mutation was identified, but considerably lower frequencies were observed in the other cohorts [5]. Moreover, it was reported that *POT1* PVs/likely PVs were present in 3.8% of 105 high-risk melanoma families from the UK, Netherlands, and Australia, making *POT1* the second most prevalent high-penetrance melanoma susceptibility gene in these populations after *CDKN2A* [6]. On the other hand, no potentially deleterious *POT1* variants were discovered in a Dutch cohort of 451 familial melanoma families, indicating their extremely low frequency in the Dutch population [11]. Similarly, in an Austrian cohort, 53 *POT1* variants were identified across a broader study population; however, only eight of those were exclusive to high-risk melanoma cases, further emphasizing the scarcity of *POT1* PVs, even among individuals with a predisposition to melanoma [12]. These findings imply that *POT1* may be a more prominent contributor to melanoma susceptibility in certain populations. However, the observed differences in prevalence could be attributed to variations in the study design, genetic background, and the inclusion criteria used for defining familial melanoma. The Spanish study inclusion criteria were less stringent, including families with at least two melanoma cases in first- or second-degree relatives [9], compared to our study requiring at least three melanomas or two FDRs with melanoma if one was diagnosed before the age of 55. The lower *POT1* PVs frequency in the Swedish cohort, despite the more stringent selection criteria, could reflect a more complex etiology of melanoma in these families, potentially related to the pigmentation phenotype and sun exposure patterns among family members [34]. Our results are derived from one region, but similar to other capital cities, Stockholm – due to urbanization and a steady influx from various regions across Sweden – consists of a heterogeneous population and could, to an extent, represent the nationwide prevalence of *POT1*.

Consistent with previous studies linking *POT1* aberrations to an increased tumor burden [5, 35], early-onset melanoma and multiple primary tumors were observed in our *POT1*-carrier family. One noteworthy feature in our cohort was the spitzoid morphology identified in the two of three invasive melanoma cases in the carriers. These tumors were therefore classified as BRAF-mutated melanomas with spitzoid features, rather than Spitz melanomas, which are molecularly defined by typical Spitz-associated gene fusions (*ALK*, *ROS1*, *NTRK1-3*, *RET*, *MET*, *BRAF*, and *MAP3K8*) or by *HRAS* activating point mutations [36, 37]. The presence of spitzoid features has also been reported in larger studies, in a high proportion of familial melanomas from individuals carrying germline variants in *POT1* and other telomere maintenance genes, suggesting that dysfunctional telomere maintenance may contribute to spitzoid differentiation in melanocytic tumors [32, 33]. Therefore, our findings further support melanoma with spitzoid features as a defining trait within the *POT1* familial cancer syndrome.

In the identified family with the p.His226Asn variant, a notable observation was the occurrence of an invasive melanoma with early onset in a confirmed non-carrier. This finding was verified through repeat genetic testing following a second blood sample, which again confirmed the absence of the variant. However, several other factors suggest that the identified *POT1* variant in this family was disease-associated, including the in-silico analyses of the variant, the strong family history with multiple cases of melanoma (with early onset, spitzoid features, and multiple primaries), and several other tumors in the pedigree, including those frequently described in *POT1* families (brain and connective tissue tumors). The non-complete segregation in this family was therefore interpreted as a likely phenocopy. Phenocopies have also been described in the setting of melanoma families with *CDKN2A* mutation and have been attributed to other risk-modifying genes and ultraviolet exposure patterns that can affect melanoma penetrance in carriers and result in a higher melanoma risk in non-carriers [38].

Previously published studies have described telomere elongation in patients carrying a *POT1* PV, compared to those with wild-type (WT) *POT1* [5], whereas in this study, no significant difference in telomere length was distinguished in the *POT1*-carriers compared to other melanoma cases or healthy controls. One possible explanation could be the low power of the telomere length analysis as it was performed on only two of the carriers (the third confirmed carrier was not available for this analysis). Of these two, one was in the seventh decade of life, and the other in the third decade at the time the blood for the analysis was collected, and it is possible that telomere length differences between *POT1* carriers and non-carriers could become more prominent with age. Additionally, we only studied the average telomere length and not the telomere length heterogeneity, which has also been observed to be more pronounced in *POT1* carriers, with some telomeres being excessively long while others remain short. Furthermore, the analyses were exclusively done from blood-derived DNA, which may not reflect the telomere status in the tumor. A more comprehensive analysis of telomere length in tumor-derived DNA might provide more information about the telomere-related pathogenicity of this variant.

Given the rarity of *POT1* germline PVs and the limited data available, the lifetime cancer risk for individuals with *POT1*-carriers remains unclear. Moreover, evidence supporting the benefit of surveillance for early cancer detection in these patients is currently insufficient. Although a recent study proposed surveillance strategies similar to those for Li-Fraumeni Syndrome, including whole-body MRI, there is no firm evidence that *POT1*-carriers face comparable risks or would benefit similarly [39]. Until recently, no peer-reviewed national or international guidelines existed for cancer surveillance in individuals with *POT1* PVs. More recently, a UK panel of experts from multiple specialties reviewed available evidence and reached a consensus on risk estimates and national clinical guidelines for managing individuals with *POT1* PVs. The surveillance recommendations, in relation to cutaneous melanoma, include baseline full skin examination at diagnosis by a dermatologist for all individuals with *POT1* PVs (age 18 or over), detailed discussion about symptom awareness and education on how to self-examine, decision on further surveillance to be made by a dermatologist based on individual’s phenotype and risk factor profile, and consideration of surveillance every 3–6 months for high-risk individuals tailored based on the individual and family. Regarding the other described *POT1*-associated tumors, surveillance is not, currently, routinely recommended, though in case of a strong family history, a cardiac or a brain MRI should be considered, for the early detection of angiosarcoma/cardiac angiosarcoma or glioma, respectively [40].

## Conclusion

Our study reveals that *POT1* aberrations are not a major contributor to hereditary melanoma in Sweden. However, the identification of a potentially *POT1* PV in a family diagnosed with multiple early-onset melanomas underscores the need for continued genetic screening in hereditary melanoma cases, especially when *CDKN2A*, *CDK4*, and *BAP1* aberrations are absent. The findings of this study contribute to the growing understanding of *POT1* variants’ role in familial melanoma. Considering the reports on *POT1* internationally and our results from a relatively small sample of melanoma families in this study, we recommend that *POT1* testing should be considered on a case-by-case basis, specifically in families with a strong family history of melanoma with early-onset, multiple primaries, and spitzoid features, co-existing with other *POT1*-related tumors. Furthermore, the development of a patient information leaflet for individuals carrying *POT1* PVs and their relatives is considered of essence, in order to raise patients’ awareness and explain their lifetime cancer risks compared with the general population. Such a resource can empower informed dialogue between patients and healthcare professionals about potential health concerns.

## Data Availability

The data that support the findings of this study are available from the corresponding author upon reasonable request.
